# Supplementary figures and images for: The Transformative Potential of Large Language Models in Mining Electronic Health Records Data: Content Analysis
Source: JMIR Med Inform. 2025 Jan 2;13:e58457. doi: 10.2196/58457 (PMC11739723; doi:10.2196/58457)

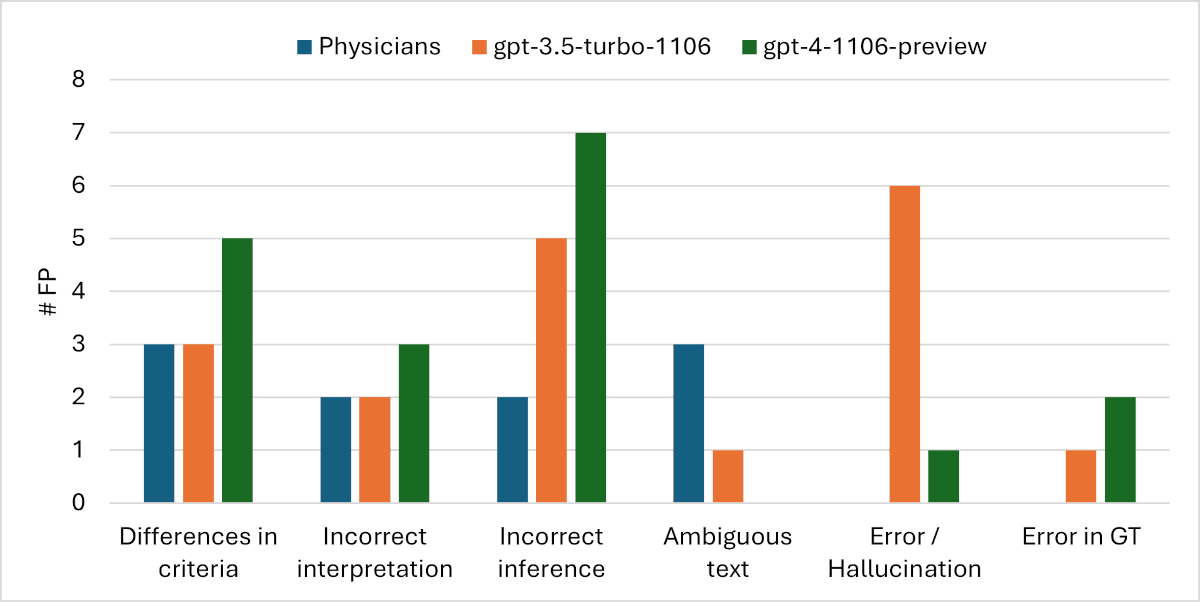

Supplement: Multimedia Appendix 1 [file medinform_v13i1e58457_app1.png]

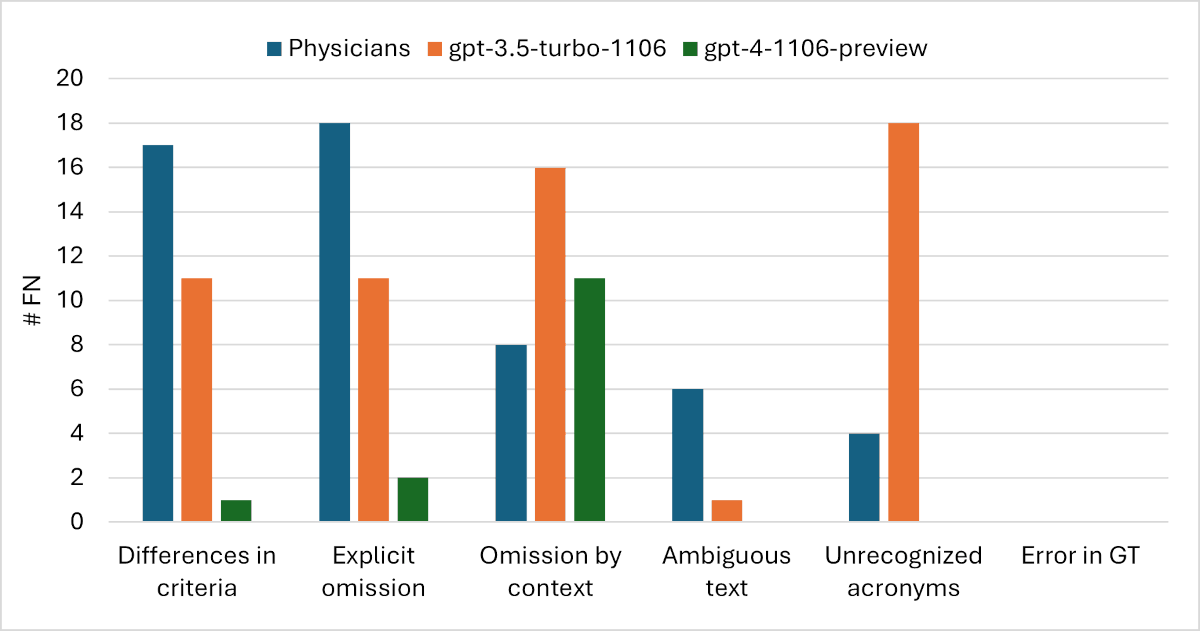

Supplement: Multimedia Appendix 2 [file medinform_v13i1e58457_app2.png]
